# Effect of miniscrew insertion angle in the maxillary buccal plate on its clinical survival: a randomized clinical trial

**DOI:** 10.1186/s40510-021-00370-8

**Published:** 2021-08-02

**Authors:** Amin Golshah, Kimia Gorji, Nafiseh Nikkerdar

**Affiliations:** 1 Department of Orthodontic, School of Dentistry, Kermanshah University of Medical Sciences, Kermanshah, Iran; 2 Students Research Committee, School of Dentistry, Kermanshah University of Medical Sciences, Kermanshah, Iran; 3 Department of Oral and Maxillofacial Radiology, School of Dentistry, Kermanshah University of Medical Sciences, Kermanshah, Iran

**Keywords:** Miniscrew, Orthodontic treatment, Clinical survival, Periotest, Visual analog scale

## Abstract

**Introduction:**

This study sought to assess the effect of miniscrew insertion angle (vertical and oblique) on its clinical survival under shearing forces in orthodontic patients undergoing canine retraction.

**Materials and methods:**

In this split-mouth randomized controlled clinical trial, 50 miniscrews were placed bilaterally in 25 patients with 45° and 90° insertion angles relative to a line perpendicular to the occlusal plane distal to the maxillary first premolar extraction site. Allocation of insertion angles to the right/left side was random using the Random Allocation Software. The patients, clinician, and statistician were blinded to the allocation of miniscrews to the side of jaw. The patients were followed-up monthly for 6 months. The primary outcome was the clinical survival of miniscrews, which was evaluated at each follow-up session. The secondary outcomes were the miniscrew stability based on the Periotest value (PTV) and the level of pain experienced by patients at 1, 12, and 24 h, and 7 days after miniscrew placement using a visual analog scale (VAS). Data were analyzed using paired t-test, repeated measures ANOVA, and McNemar’s test.

**Results:**

The clinical survival rate of miniscrews placed at 90° and 45° angles was 76% and 88%, respectively. This difference was not statistically significant (*P* = 0.375). No significant difference was noted between the two groups regarding the PTV or the pain score either (*P* > 0.05).

**Conclusion:**

Clinically, the insertion angle of miniscrews (90° versus 45° relative to a line perpendicular to the occlusal plane) has no significant effect on the miniscrew survival rate or stability during orthodontic treatment.

**Trial registration:**

This trial was registered at www.irct.ir (IRCT20190901044659N1).

**Protocol:**

The protocol was published after trial commencement.

## Introduction

Anchorage in orthodontics simply means prevention of unwanted tooth movements. Miniscrew placement is one suggested strategy to provide orthodontic anchorage [1]. Miniscrew placement is a minimally invasive procedure as long as the risk of contact and traumatization of tooth roots is considered. Easy insertion and removal, no dependence on patient cooperation, low cost, and insignificant postoperative pain and discomfort are among the other advantages of miniscrews [1–3]. Due to the aforementioned advantages, miniscrews are extensively used by orthodontists for tooth movement in conventional orthodontic procedures such as molar protraction, canine retraction, correction of dental midline, space closure, and distalization of maxillary molars [1, 4]. However, miniscrews are susceptible to mobility and failure during the treatment course. Several studies have evaluated the causes of miniscrew failure; however, the precise factors responsible have not been clearly identified [5–7]. The main factors responsible for miniscrew failure that have been reported so far include inflammation of bone supporting the miniscrew and the adjacent structures, inappropriate site of miniscrew insertion, poor stability due to inadequate cortical bone thickness, bone-related factors, poor oral hygiene status, and age of patients [8, 9].

The clinical survival of miniscrews depends on absence of inflammation, detectable mobility or unbearable pain, and the ability of the miniscrew to remain stable and in function under orthodontic forces [7].

The insertion angle is among the suggested factors affecting the clinical survival and primary stability of miniscrews [10–19]. The survival rate of miniscrews has been reported in many previous clinical trials; however, no consensus has been reached on the effect of insertion angle of the miniscrews on their survival rate [7, 20–22].

The available clinical trials on miniscrews have some shortcomings. For instance, the pioneers of this topic did not focus on populations with one single indication for placement or insertion site of miniscrews. Instead, many authors published data derived from all their cases with a mixture of different insertion sites, screws, and indications. However, later trials that focused, for example, on buccal miniscrews for en masse retraction in the maxilla reported higher survival rates [23–25]. Such a variation in survival rates indicates that we should focus on populations with only one indication or treatment concept for miniscrew placement.

### Specific objectives

Considering the role of different factors in clinical survival of miniscrews, the existing controversy in previous studies regarding the effect of miniscrew insertion angle on its clinical survival, and lack of proper clinical trials on this topic, this study aimed to assess the effect of miniscrew insertion angle (vertical and oblique) on its clinical survival rate under shearing forces in orthodontic patients undergoing canine retraction by measuring the miniscrew mobility and pain in the supporting structures. The primary aim was to assess the clinical survival of miniscrews, while the secondary aims were to evaluate the miniscrew stability and the level of pain experienced by patients. The null hypothesis was that the miniscrew insertion angle would have no significant effect on clinical survival or stability, or the level of pain experienced by patients.

## Materials and methods

### Trial design

This was a parallel group, split-mouth, randomized, active-controlled clinical trial with 1:1 allocation ratio. This study was approved by the ethics committee of Kermanshah University of Medical Sciences (IR.KUMS.REC.1398.694) and registered in the Iranian Registry of Clinical Trials (IRCT20190901044659N1).

### Participants, eligibility criteria, and settings

Twenty-five consecutive patients whose treatment plan included miniscrew insertion in the maxillary buccal plate for maxillary canine retraction into the maxillary first premolar extraction space under shearing orthodontic forces were recruited from a private orthodontic office from December 2019 to August 2020. The inclusion criteria were patients with skeletal class II division 1 malocclusion with molars in a full-cusp class II relationship and normal angle of the maxilla relative to the mandibular plane (25°±5°) requiring first premolar extraction and maxillary canine retraction. The exclusion criteria were periodontal disease or bone loss, gross facial asymmetry, cleft lip/palate, impacted or missing teeth at the treatment site, systemic diseases, medication intake, suboptimal frenum position, inadequate length and/or thickness of the attached gingiva, craniofacial disorders of any type, syndromes, systemic diseases, cigarette smoking, and poor oral hygiene. The extractions were performed before the miniscrew placement.

No changes to methods occurred after trial commencement.

### Interventions

**Fig. 1:**
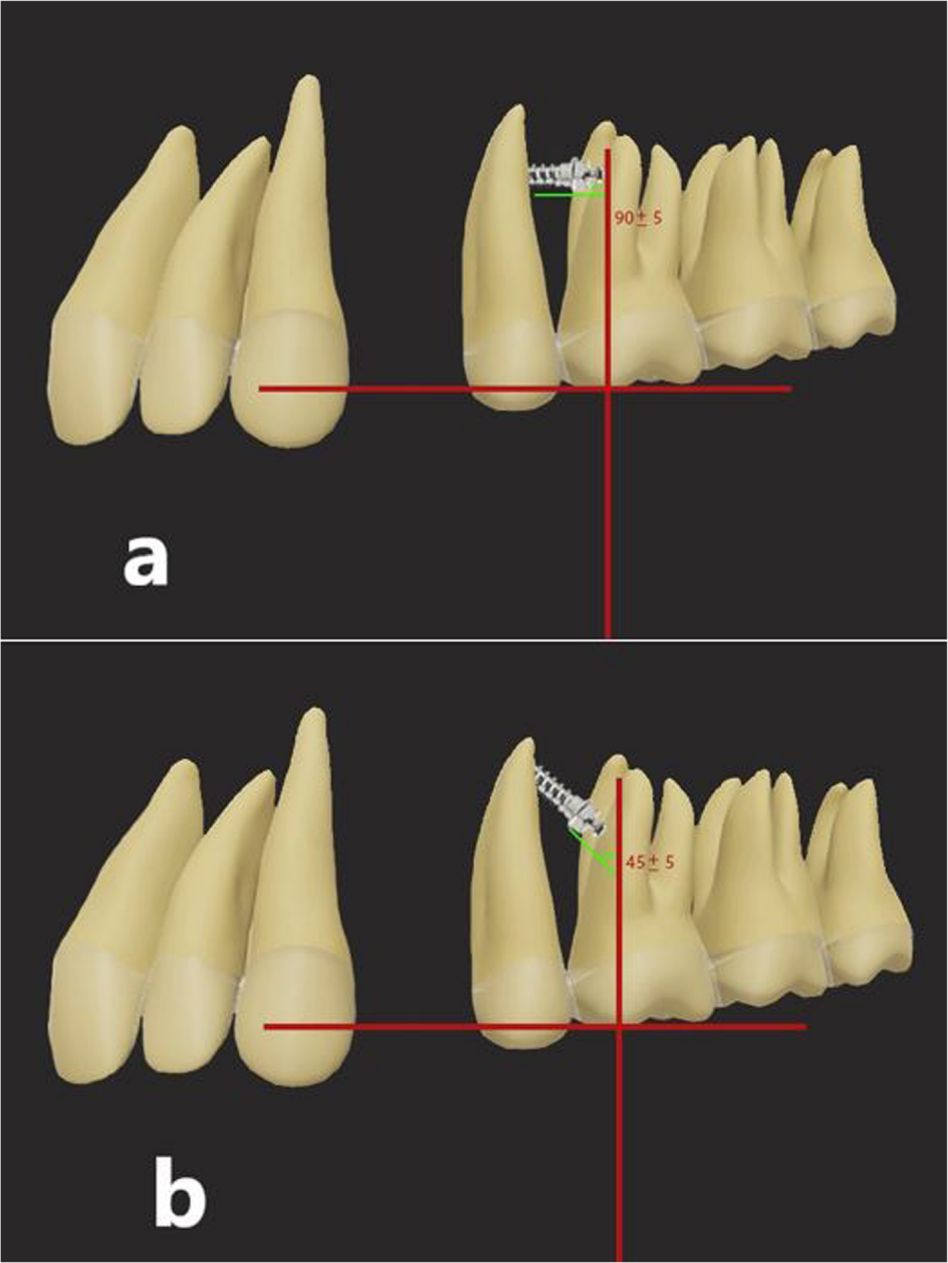
Schematic view of the insertion of miniscrews: **a** 90° insertion angle, **b** 45° insertion angle

**Fig. 2:**
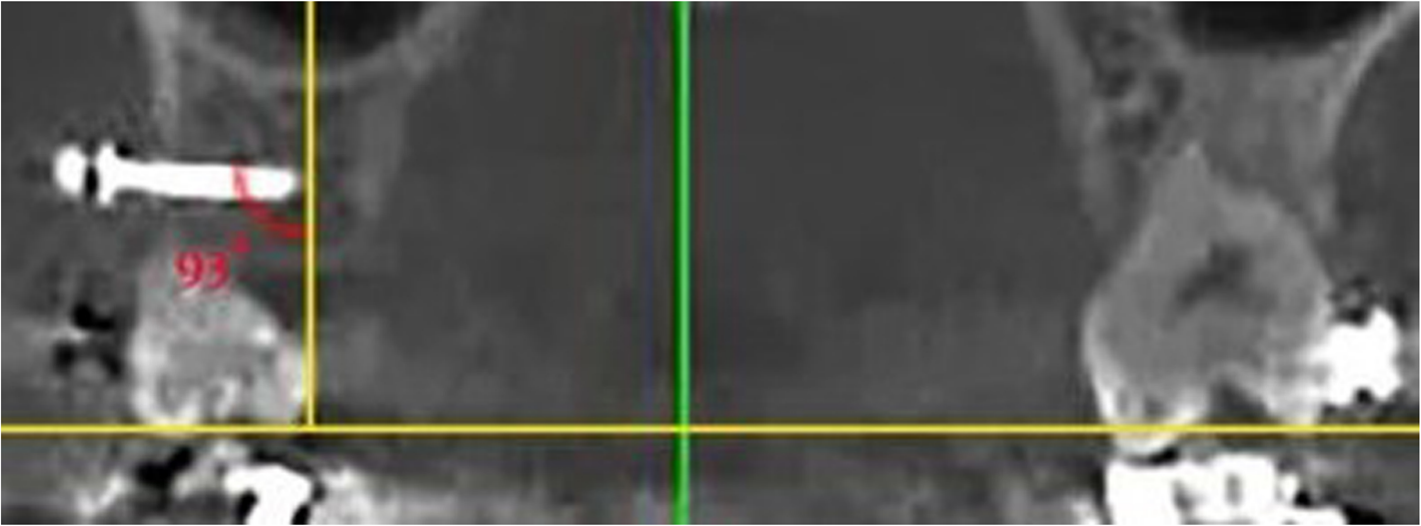
Coronal cross-sectional CBCT image for measuring the insertion angle of miniscrews

After obtaining written informed consent from the patients, the treatment was commenced by using Roth 22 brackets (Discovery, Dentaurum, Germany) and bands (American Orthodontics, USA). After leveling and aligning of the teeth by using the wire sequence of 0.016 nickel titanium, 0.017 × 0.025 nickel titanium, and 0.017 × 0.025 stainless steel, the patients were requested to rinse their mouth with 10 cc of 0.2% chlorhexidine mouthwash (Parodontax, Germany) prior to miniscrew placement. Next, local anesthesia was administered bilaterally by infiltration anesthesia using 2% lidocaine plus 1:100,000 epinephrine. The miniscrews were then placed at 45° and 90° angles relative to a line perpendicular to the occlusal plane into the attached gingiva between the maxillary second premolar and the maxillary first molar (Fig. 1). After bilateral placement of miniscrews, cone-beam computed tomography (CBCT) scans were obtained to ensure correct insertion angle of the miniscrews, no contact of miniscrews with the lingual cortical plate or the sinus wall, and adequate distance from the roots (Fig. 2). All CBCT images were obtained with 8 × 8 cm field of view, 150-μm spatial resolution, 4.19 mAs amperage, and 110 kV voltage. The CBCT data were exported in DICOM format using the NNT Viewer software version 8 (QR s.r.l, Verona, Italy). A ±5° error range was considered acceptable for the insertion angle of miniscrews; otherwise, the case would be replaced. All miniscrews were placed by the same operator (A.G.). The miniscrews used in this study (G2; Jeil, Seoul, South Korea) had 8 × 1.6 mm dimensions and were placed in the alveolar bone with an Orthonia driver (Jeil, Seoul, South Korea) with 10 Ncm torque and medium speed (rpm). The patients were instructed to rinse their mouth with 0.12% chlorhexidine mouthwash twice a day (in the morning and in the evening) for 1 week after the insertion of miniscrews. Also, they were requested to clean the area around the miniscrews after each meal using a soft toothbrush. Two weeks after the placement of miniscrews, canine retraction was started by applying 150 g force with a nickel-titanium closed coil spring (G&H orthodontics, Franklin, IN, USA) connecting the miniscrew to the hook of the canine bracket of the corresponding side. The patients were followed up monthly for a total of 6 months. At each follow-up session, after removing the spring, the stability and clinical survival of miniscrews were evaluated by the same operator (K.G.), and the spring was subjected to load application again. At each side, 150 g force with a nickel-titanium closed coil spring (G&H orthodontics, Franklin, IN, USA) was applied. At each treatment session, the coil spring was separated from its attachment area to the canine tooth and deactivated. The force was measured by a force meter, and the coil spring was reattached to the canine tooth and activated again.

### Outcomes (primary and secondary)

The primary outcome was to assess the effect of miniscrew insertion angle in the maxillary buccal plate on its clinical survival. The miniscrew clinical survival criteria were as follows:

(I) Absence of signs and symptoms of inflammation such as redness, edema, and exudate
(II) Absence of visible mobility (>1 mm) indicating that the miniscrew can be used for orthodontic anchorage
(III) Absence of unbearable pain

Presence of any of the abovementioned criteria during the follow-up period was recorded as failure in a checklist. For continuation of patient treatment, the failed miniscrews were removed and reinserted after 1 month, if required.

Assessment of the stability of miniscrews by a Periotest and level of pain experienced by patients using a visual analog scale (VAS) was the secondary outcomes of the study. There were no outcome changes after trial commencement.

### Assessment of the level of pain

The level of pain experienced by patients was measured using a 10-mm VAS at 1, 12, and 24 h, and 7 days after the placement of miniscrews. In case of presence of unbearable pain, it was recorded as failure in the checklist [26].

### Assessment of stability

**Fig. 3:**
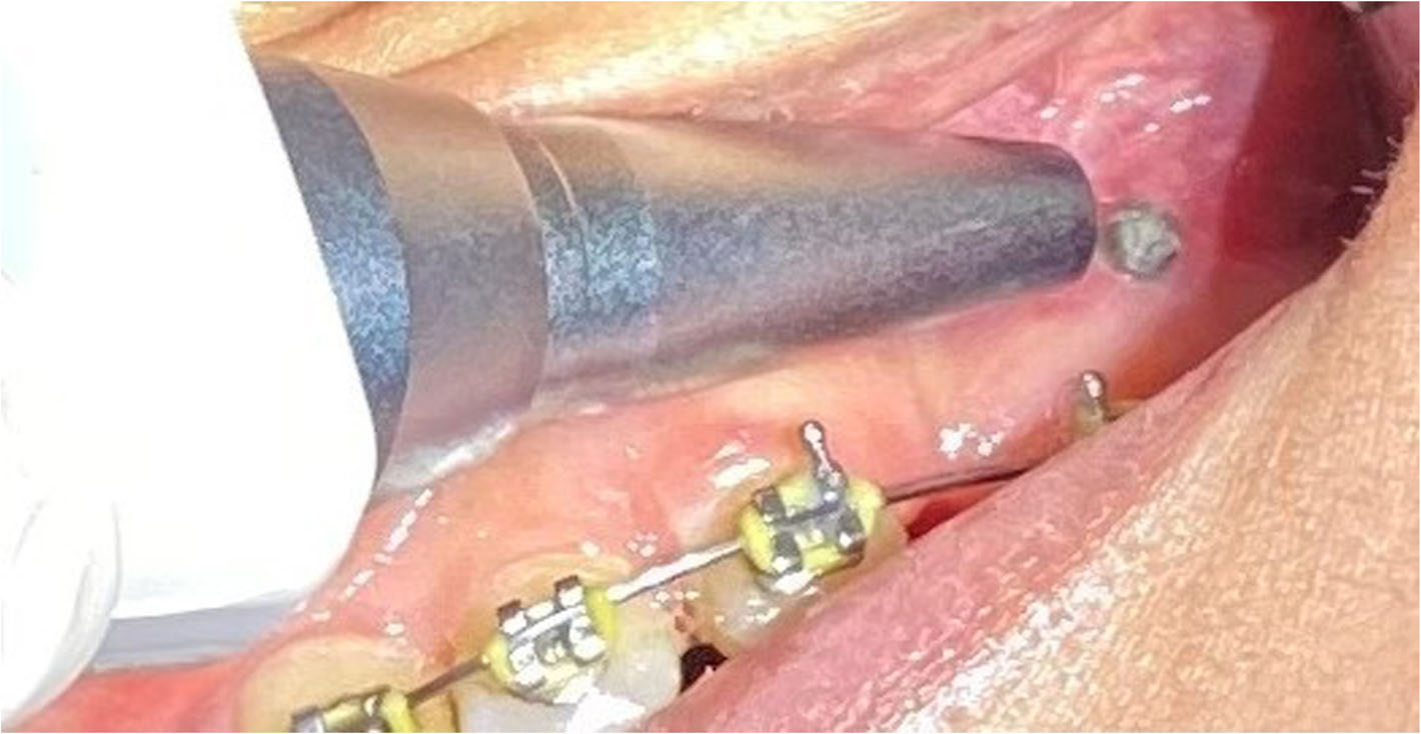
Periotest was held perpendicular to the longitudinal axis of the miniscrew to evaluate its stability

The stability of miniscrews was evaluated by a Periotest (Medizintechnik Gulden, Germany), which is an electromechanical device for assessment of stability and mobility. The value displayed on the Periotest monitor, referred to as the Periotest value (PTV), can range from −8 to +50. The smaller the PTV, the higher the stability; 0 indicates normal physiological mobility while −8 indicates maximum stability [27]. The Periotest was held perpendicular relative to the longitudinal axis of the miniscrew, and three values were recorded at each position (Fig. 3). The mean value was then calculated and recorded as the PTV [27]. The PTV values were measured on a monthly basis during the study period for 6 months.

### Sample size calculation

The minimum sample size was calculated to be 24 patients according to a previous study by Park et al. [7] assuming the survival rate of the groups with miniscrew insertion angles of 30–40° and 90° to be 0.952 and 0.852, respectively, alpha=0.05, study power of 90%, rate of dropouts to be 0.15, and accuracy (d) of 0.2.

### Randomization

Only patients who required bilateral maxillary miniscrews were enrolled in this split-mouth randomized clinical trial. The allocation of miniscrew insertion angles to the left and right maxillary quadrants was random and determined by using the Random Allocation Software (version 2.0; Isfahan, Iran) in 1:1 distribution ratio and equal numbers. Following the random sequence generation, random numbers were allocated to each patient in sealed opaque envelopes. Before the miniscrew placement, the envelope was opened to disclose the angle of miniscrew placement for each maxillary quadrant of the respective patient. The chief of the department, who had no financial, research, or publishing interest in this study, performed the random number generation, allocation, concealment, and implementation.

### Blinding

Allocation of miniscrew angulations to the side of the jaw was random in a triple-blind manner. The patients, the clinician who evaluated the stability and clinical survival of miniscrews (K.G), and the statistician were blinded to the allocation of miniscrews to the side of the jaw.

### Statistical analysis

The Kolmogorov-Smirnov test was applied to assess the normality of data distribution. Since the data were normally distributed, repeated measures ANOVA was used to analyze the changes in PTV over time. The Wilcoxon signed-rank test was applied to compare the pain score between the two quadrants while the Friedman test followed by the Dunn-Bonferroni test was used to analyze the changes in pain score over time. The McNemar test was used to compare the clinical survival of 45° and 90° miniscrews as well as the odds ratios at 95% confidence interval. All statistical analyses were carried out using SPSS version 25 (SPSS Inc., IL, USA) at 0.05 level of significance.

### Method error analysis

For assessment of the reliability of the PTV measurements, 30% of the cases were remeasured by a highly skilled dental student (K.G.) with the same method. The minimum interclass correlation coefficient was calculated to be 0.933.

## Results

### Participant flow and baseline data

**Fig. 4:**
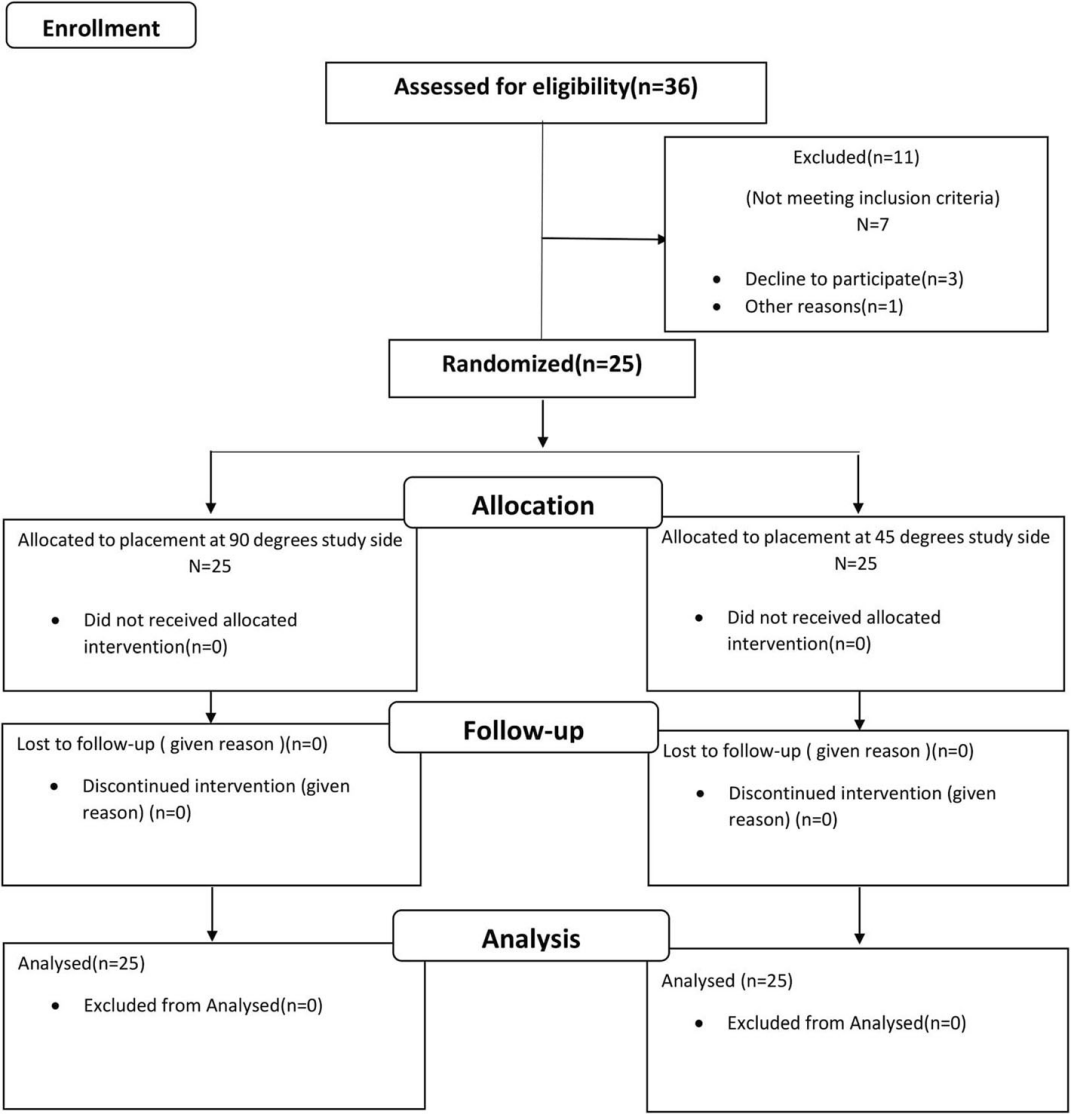
Flow diagram of the study according to the Consolidated Standards of Reporting Trials

A total of 36 patients were eligible for participation in this trial. Of these, 11 were excluded since they did not meet the inclusion criteria or were not willing to participate in the study. Thus, this trial enrolled 25 participants including 9 males and 16 females. Figure 4 shows the flow diagram of the study according to the Consolidated Standards of Reporting Trials. One miniscrew was placed in each quadrant of the maxilla (a total of 50).

### Primary outcome

The clinical survival rate of miniscrews placed at 90° and 45° angles was 76% and 88%, respectively, with no significant difference (McNemar test, *P* = 0.375). Of all, 6 miniscrews (24%) placed at 90° angle and 3 miniscrews (12%) placed at 45° angle failed.

### Secondary outcomes

Table 1 compares the PTV of 45° and 90° miniscrews over time. No significant difference was noted in the PTV of 45° and 90° miniscrews at any time point (*P* > 0.05). Table 2 compares the median pain scores between the 45 and 90° miniscrew groups at different time points. The median pain score was not significantly different between the two groups at any time point (*P* > 0.05). However, within-group comparisons revealed a significant change in the pain score of patients in the 90° miniscrew group at different time points (*P* < 0.001), such that the maximum pain score was recorded after 1 h and then decreased until day 7. Similarly, a significant change was noted in the pain score of patients in the 45° miniscrew group at different time points (*P* < 0.001) such that the maximum pain score was recorded after 1 h and then decreased until day 7.

**Table 1:** Comparison of the PTV of 45° and 90° miniscrews over time

Time	90°		45°		95% confidence interval		*P*-value^†^
	Mean	SD	Mean	SD			
					Lower	Upper	
At 1 month	4.07^a^	7.76	3.72^a^	9.19	−4.28	4.71	0.923
At 2 months	4.64^a^	7.94	4.64^a^	9.76	−4.82	4.42	0.929
At 3 months	5.66^a^	6.96	4.04^a^	7.85	−2.63	5.56	0.466
At 4 months	5.20^a^	6.25	4.07^a^	5.89	−1.29	2.90	0.432
At 5 months	4.71^a^	6.83	3.68^a^	5.66	−2.67	4.13	0.656
At 6 months	2.95^a^	5.06	6.01^a^	5.13	−8.12	1.40	0.150
*P*-value^‡^	0.104		0.160				

^†^Paired t-test

^‡^Repeated measures ANOVA followed by LSD test

Means with the same superscript letters are not significantly different (*P* > 0.05)

**Table 2:** Comparison of the median pain scores between the 45 and 90° miniscrew groups at different time points

Time	90°		45°		*P*-value^†^
	Median	IQR	Median	IQR	
At 1 h	4^c^	1	4^c^	2	0.511
At 12 h	2^b^	1	2^b^	1	0.595
At 24 h	1^ab^	2	1^ab^	1	0.108
At 7 days	0^a^	0	0^a^	0	0.317
*P*-value^‡^	<0.001		<0.001		

^†^Wilcoxon signed-rank test

^‡^Friedman test followed by Dunn’s multiple comparisons test (significant values have been adjusted by the Bonferroni correction for multiple tests)

Medians with the same superscript letters are not significantly different (*P* > 0.05)

*IQR* interquartile range

### Harms

No serious harm was observed after miniscrew placement other than pain.

## Discussion

The primary stability of miniscrews is achieved by the mechanical interlocking of their threads in the cortical bone. However, even in case of optimal primary stability, application of excessive loads to the surrounding bone may interfere with the physiological remodeling of the miniscrew surface and bone, and lead to failure [10]. The failure rate of miniscrews is variable in the literature. For example, Papageorgiou et al. [28] reported a failure rate of 13.5% while this rate was 14.5% in a study by Motoyoshi et al. [29] and 14.2% in a study by Watanabe et al. [22].

The survival rate of miniscrews reported in the literature is often over 80% [30–34]. The variable range of clinical survival rate of miniscrews may be due to different survival and failure criteria in different studies and variability in study populations in terms of age, gender, anatomical position of miniscrew, type of malocclusion, and oral hygiene status [35]. Also, miniscrew-related factors such as type, length, and diameter of miniscrews are variable [36]. Moreover, the magnitude and duration of load application and type of orthodontic movement also vary in different studies [37].

This study assessed the effect of miniscrew insertion angle (vertical and oblique) on its clinical survival under shearing forces in orthodontic patients. Clinically, the insertion angle of miniscrews (90° versus 45°) had no significant effect on the miniscrew survival rate or stability during orthodontic treatment, or the level of pain experienced by patients. Thus, the null hypothesis of the study was accepted. Optimal stability is one of the survival criteria of miniscrews [10]. Based on this fact, some in vitro studies investigated the effect of insertion angle of miniscrews on their stability and reported controversial results. Some studies showed that oblique insertion of miniscrews decreased their stability [14–16, 38–40], while some others reported that oblique placement of miniscrews increased their stability [10–13, 41–44]. The results of these studies were different from our findings, which may be due to their in vitro design and poor generalizability of their results to the clinical setting. Recently, an in vitro study evaluated the effect of size, technique of placement, and insertion angle of miniscrews on primary stability of mini-implants. They discussed that the primary stability of miniscrews depended on the density of the cancerous bone. They found that angulation of miniscrew in high-density bone decreased the primary stability, but this was not the case for the low-density bone. Thus, they recommended assessment of cancellous bone density prior to miniscrew placement [45].

In line with our results, some previous clinical trials demonstrated that the insertion angle had no significant effect on miniscrew survival [7, 20–22]. The main shortcoming of such trials was that they did not focus on populations with uniform insertion sites, screws, or indications, whereas the present trial only focused on skeletal class II division 1 malocclusion patients with molars in a full-cusp class II relationship and normal angle of the maxilla relative to the mandibular plane (25°±5°). The patients required first premolar extraction and maxillary canine retraction by using bilateral maxillary miniscrews placed in the maxillary buccal plate. The manufacturing company and dimensions of all inserted miniscrews were the same (G2; Jeil, Seoul, South Korea, 8 × 1.6 mm). Also, the miniscrews were loaded by equal shearing orthodontic forces in all patients (150 g). By doing so, the confounding effect of all these variables on the results was eliminated.

In this study, Periotest was used to assess the stability of miniscrews, which showed that the miniscrew insertion angle had no significant effect on the PTV. Optimal clinical application of Periotest for assessment of miniscrew stability was first confirmed in an in vitro study [46]. The authors reported that the insertion torque was correlated with the PTV, such that the higher the insertion torque in bone, the lower the PTV and the higher the primary stability would be [46]. Watanabe et al. [47] confirmed this finding and suggested the PTV, the amount of insertion torque, and the cortical bone thickness as the criteria for clinical stability and survival prediction of miniscrews. According to Atsumi et al. [48], Periotest is a non-invasive quantitative tool with maximum efficacy for measurement of the primary stability of dental implants.

Measurement of pain by VAS in this study revealed that the pain score was maximum at 1 h after miniscrew placement and decreased over time in both groups. Mirhashemi et al. [49] in their clinical study evaluated the pain score following miniscrew insertion in orthodontic patients and reported maximum pain at 1 h after miniscrew placement; the pain score had a descending trend after that for 7 days. Kuroda et al. [1] reported maximum pain at 1 h after miniscrew placement and its decreasing trend for up to 7 days. The abovementioned results support our findings. Łyczek et al. [50] evaluated the level of pain and inflammation following miniscrew insertion with 45° angle and reported mild pain and discomfort after 1 day.

In total, since none of the tested insertion angles of miniscrews had any superiority over the other, clinicians can place the miniscrews vertically or obliquely based on the clinical and anatomical patient conditions, position of the teeth and roots, and type of applied force, and this decision would not affect the clinical survival of miniscrews.

Future clinical trials with larger sample size are required to assess the effect of other types of orthodontic forces in different miniscrew insertion angles on their clinical survival rate. Moreover, a recent study reported computer-guided placement of mini-implants [51], which calls for further studies regarding the clinical survival and possible complications of miniscrews placed by this technique.

### Limitations

Small sample size and not measuring the level of pro-inflammatory cytokines in the gingival crevicular fluid were among the limitations of this study. Future studies with larger sample size and multi-center design are required to measure the level of pro-inflammatory cytokines in the gingival crevicular fluid following miniscrew placement at different angles. Also, the miniscrews were loaded 2 weeks after placement in this study, which may not be an ideal time since at this moment primary stability probably decreases while secondary stability is not yet present. Future studies are required to assess the mini-screw stability immediately after loading [52].

### Generalizability

The generalizability of the current results might be limited because this study was undertaken in one center by one clinician (A.G.) experienced in placing miniscrews.

## Conclusion

According to the results, the clinical survival rate and the stability of miniscrews and the level of postoperative pain and discomfort experienced by patients were not significantly different when oblique (45°) and vertical (90°) insertion angles were compared.

## Data Availability

The datasets generated and/or analyzed during the current study are not publicly available because of the privacy of patients but are available from the corresponding author on reasonable request.

## Abbreviations

PTV: Periotest value
VAS: Visual analog scale
CBCT: Cone-beam computed tomography
